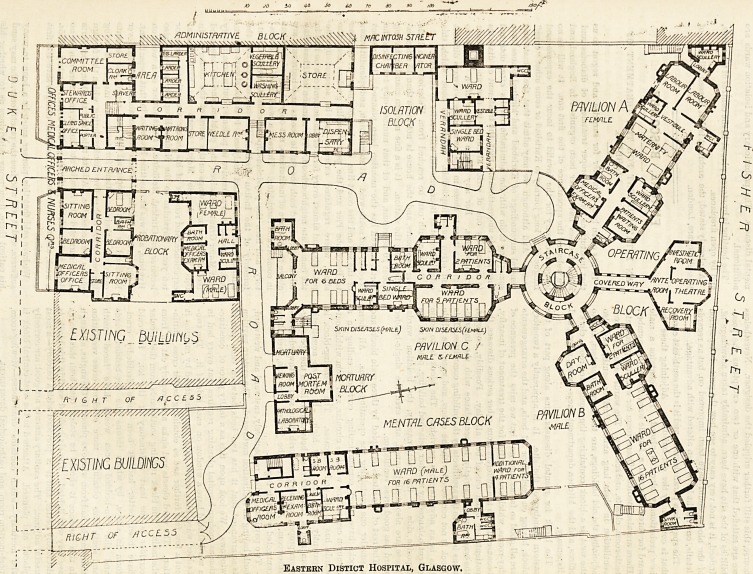# The Eastern District Hospital, Glasgow

**Published:** 1903-06-20

**Authors:** 


### THE EASTERN DISTRICT HOSPITAL, GLASGOW.

THE architect who has to design a hospital for a large town, where the land at disposal for a site is often limited in extent and always high in market value, has a difficult task given to him for solution, and this problem was certainly

EASTERN DISTRICT HOSPITAL, GLASGOW.

put before Mr. Hessell Tiltman when he arranged the various blocks which constitute the Glasgow Eastern District Hospital. Part of the site was already occupied by buildings which it was deemed advisable to retain and, of course, this still further crippled the designer. Nevertheless we are satisfied that with one or two exceptions the possibilities of the site have been admirably taken advantage of. Indeed, it is not very easy to see how the general disposition of the blocks could have been improved on, but as regards details, one might have been altered with advantage.

The space is a parallelogram, and it is the south-east corner that is covered by the old buildings. Adjoining these the new probationary block is placed. This contains a male ward for three beds and a female ward for a like number. There are also medical officers' room for examining patients, bath-room, closets, and scullery. The wards have good cross ventilation in their long axis. Further south, and also adjoining the old buildings, are the nurses' quarters, and to the west is a block containing the committee-room, clerk's offices, and the kitchen department, mess-rooms, stores, and dispensary.

The block for mental diseases runs along part of the eastern boundary of the site. It is apparently a two-story block, having on each floor 22 beds, and the arrangements are all good so far as they go; but we miss the presence of day-rooms, which are essential for the treatment of insanity, because, unlike the sufferers from other serious diseases, the patients are often out of bed all day. Possibly, however, the wards may be strictly in accordance with the instructions issued to the architects by the building committee. This block is not connected with the others, and upon the whole this is an advantageous provision. The block is to some extent balanced by another detached building, placed at the opposite side of the plot, namely, the isolation wards. These are also well planned, and even the single-bedded ward has good cross ventilation.

The rest of the site is occupied by three main blocks and a smaller one containing the operating-rooms. All these blocks have short communicating corridors ending in a common central one which is circular, and from within outwards has a lift and staircases. The operating-block is apparently only one story high. It has an anæsthetic room and a recovery room between which is the operating-room proper, having good north light, and southwards is the ante-room. As far as can be judged from plan, everything connected with this is good.

Two pavilions strike, respectively, eastward and westward, both having an inclination northward. The eastern pavilion is so arranged that it has two beds between each pair of windows. We look upon this as a faulty piece of construction as modern hospital planning requires that every bed in a ward should have a window on each side of it. The two-bedded ward, also, would have been improved by running it out a few feet further and so obtaining better cross ventilation. The ward closets and sinks are properly cut off from the main. The block running southwards from the circular corridor is the least satisfactory of the lot. It would have been better had it been more decidedly cruciform in shape, by which means the five-bedded wards and the two-bedded ones would have faced north and south thus obtaining more efficient cross ventilation than can be had from windows placed in the angles, although in this instance it should be stated that both these wards are very well lighted. The six-bedded ward in this block is decidedly better, and it is provided with a balcony—a most useful adjunct. The sanitary annexes are good, are correctly placed and planned. Attached to the west-central block is the mortuary—which contains dead-house—viewing-room, post-mortem room, and pathological department.

The hospital is intended for acute cases arising in its own neighbourhood. The total accommodation is for 244 beds. Of these 108 are medical, 50 are surgical, 14 for skin diseases, 10 for children's diseases, 4 for maternity cases, and 44 for mental diseases. The probationary and isolation wards are additional.

The heating is by stoves, placed in the centres of the wards, and by open fireplaces.

As already stated, the architect is Mr. Hessell Tiltman, of Russell Square, London.

## Figures and Tables

**Figure f1:**